# Differential Expression of Matrix Metalloproteinases 2, 9 and Cytokines by Neutrophils and Monocytes in the Clinical Forms of Chagas Disease

**DOI:** 10.1371/journal.pntd.0005284

**Published:** 2017-01-24

**Authors:** Nayara I. Medeiros, Rafaelle C. G. Fares, Eliza P. Franco, Giovane R. Sousa, Rafael T. Mattos, Ana T. Chaves, Maria do Carmo P. Nunes, Walderez O. Dutra, Rodrigo Correa-Oliveira, Manoel O. C. Rocha, Juliana A. S. Gomes

**Affiliations:** 1 Laboratório de Imunologia Celular e Molecular, Centro de Pesquisa René Rachou, FIOCRUZ, Belo Horizonte, Minas Gerais, Brasil; 2 Laboratório de Biologia das Interações Celulares, Departamento de Morfologia, Instituto de Ciências Biológicas, Universidade Federal de Minas Gerais, Belo Horizonte, Minas Gerais, Brasil; 3 Faculdade de Medicina, Programa de Pós-graduação em Ciências da Saúde: Infectologia e Medicina Tropical, Universidade Federal de Minas Gerais, Belo Horizonte, Minas Gerais, Brasil; 4 Instituto Nacional de Ciência e Tecnologia em Doenças Topicais (INCT-DT), Belo Horizonte, Minas Gerais, Brasil; 5 NUPEB, Universidade Federal de Ouro Preto, Ouro Preto, Minas Gerais, Brasil; Yeshiva University Albert Einstein College of Medicine, UNITED STATES

## Abstract

Dilated cardiomyopathy, the most severe manifestation in chronic phase of Chagas disease, affects about 30% of patients and is characterized by myocardial dysfunction and interstitial fibrosis due to extracellular matrix (ECM) remodeling. ECM remodeling is regulated by proteolytic enzymes such as matrix metalloproteinases (MMPs) and cytokines produced by immune cells, including phagocytes. We evaluated by flow cytometry the expression of MMP-2, MMP-9, IL-1β, TNF-α, TGF-β and IL-10 by neutrophils and monocytes from patients with indeterminate (IND) and cardiac (CARD) clinical forms of Chagas disease and non-infected individuals (NI), before and after *in vitro* stimulation with *Trypanosoma cruzi* antigens. Our results showed an important contribution of neutrophils for MMPs production, while monocytes seemed to be involved in cytokine production. The results showed that neutrophils and monocytes from IND and CARD patients had higher intracellular levels of MMP-2 and MMP-9 than NI individuals. On the other hand, *T*. *cruzi* derived-antigens promote a differential expression of MMP-2 and MMP-9 in patients with Chagas disease and may regulate MMPs expression in neutrophils and monocytes, mainly when a cardiac alteration is not present. Our data also showed that in the presence of *T*. *cruzi* derived-antigens the production of cytokines by neutrophils and monocytes, but mainly by monocytes, may be intensified. Correlation analysis demonstrated that MMP-2 had a positive correlation with IL-10 and a negative correlation with IL-1β, whereas MMP-9 showed a negative correlation with IL-10. We also observed that IND patients presented a greater percentage of high producer cells of regulatory molecules when compared to CARD patients, indicating a different pattern in the immune response. Our data suggest that MMPs and cytokines produced by neutrophils and monocytes are important contributors for cardiac remodeling and may be an interesting target for new biomarker research.

## Author Summary

Chagas disease, also known as American trypanosomiasis, is a neglected parasitic disease caused by the protozoan *Trypanosoma cruzi*. After the acute phase, a chronic debilitating disease is established and different clinical forms may develop. Dilated cardiomyopathy is the most severe alteration, affecting about 30% of patients, and is characterized by myocardial remodeling and interstitial fibrosis due to changes in the extracellular matrix (ECM). Immune response is important to determine clinical outcome and/or disease establishment; however, it is not completely known how it participates in cardiac remodeling. Our study aims to understand the capability of neutrophils and monocytes from IND and CARD patients to produce matrix metalloproteinases and cytokines in response to *in vitro* stimulation with parasite-derived antigens. The present study showed that neutrophils and monocytes may contribute for inflammation and fibrosis in Chagas' cardiomyopathy through the production of MMPs and cytokines, respectively.

## Introduction

Chagas disease, a disease caused by the infection with *Trypanosoma cruzi*, was described by Carlos Chagas [1] over a century ago and is still a major public health problem in Latin America. There is a potential threat to other non-endemic countries, mainly in the United States, Europe and the Western Pacific region, due to the growing number of imported cases [2–4]. Currently, there are approximately 10 million people chronically infected worldwide [5]. There is no vaccine or cure available.

In the chronic phase of Chagas disease, the majority of infected individuals present the indeterminate clinical form (IND), with no overt disease [6, 7]. However, about 30% of infected individuals develop the cardiac clinical form (CARD), which presents a wide spectrum of cardiac involvement, ranging from mild to dilated chronic cardiomyopathy, the most severe manifestation of human Chagas disease with high mortality rate. Chagas' cardiomyopathy is characterized by an intense inflammatory response with destruction of cardiac fibers in the inflammatory focus and damage of the nerve plexus, leading to changes in extracellular matrix (ECM) and the development of myocardial fibrosis with myocardial dysfunction and consequent heart failure [7–9].

ECM synthesis and degradation are controlled by proteolytic enzymes such as matrix metalloproteinases (MMPs) and cytokines [10, 11]. MMPs are important enzymes involved in many physiological and pathological conditions through the degradation of ECM molecules (e.g., collagen, laminin, and fibronectin) and release of cryptic epitopes from ECM [10, 12–15]. These enzymes have a wide spectrum of biological functions, including the activation of several biomolecules, i.e. cytokines, hormones and chemokines [10, 12]. Among MMPs, the gelatinases MMP-2 and MMP-9 have been associated with cardiovascular disorders [14, 16–20], including Chagas' cardiomyopathy [21, 22].

The presence of MMPs and cytokines in heart's inflammatory infiltrate seems to have a pivotal role in the pathogenesis of cardiac morbidities [23–25]. Many cell types can produce MMPs and cytokines; however, neutrophils are the main immune cellular source of MMPs [26] and monocytes are known to be capable of producing cytokines [27–31]. These innate immunity cells begin inflammation response and fibrosis in heart through the continuous antigen stimulation, which can be by pathogen-associated molecular patterns (PAMPs) or by damage-associated molecular patterns (DAMPs) [32].

Several studies seek to understand the mechanisms linked to the development of severe forms in the chronic phase of Chagas disease. The role of innate immunity, mainly the participation of neutrophils and monocytes in the Chagas' cardiomyopathy, is yet to be explored. The study of MMPs, cytokines and innate immunity cells may be important for the identification of patients who are at risk of Chagas disease progression before the cardiac damage, with impact on patient management. In this work, we evaluated the intracellular levels of MMP-2, MMP-9 and the cytokines IL-1β, TNF-α, TGF-β and IL-10 in neutrophils and monocytes from patients with IND and CARD clinical forms of Chagas disease and non-infected (NI) individuals, before (*ex vivo* condition) and after *in vitro* stimulation with *T*. *cruzi* derived antigens.

## Materials and Methods

### Study population

Eighteen patients agreed to participate in this study and were identified and selected at the Referral Outpatient Center for Chagas Disease in *Hospital das Clínicas* of *Universidade Federal de Minas Gerais* (UFMG), Brazil. Serology for Chagas disease was determined by two or more tests (indirect immunofluorescence, ELISA or indirect hemagglutination) and patients were considered infected when at least two different tests were positive. Patients who consented to participate in this study were enrolled in a prospective cohort study initiated 17 years ago and followed annually for clinical variables specific for Chagas disease as previously described [6, 7] and are not under any type of treatment against *T*. *cruzi* infection. The patients infected with *T*. *cruzi* were grouped as indeterminate (IND) and cardiac (CARD) patients as previously reported [6, 7]. The IND group (n = 8) included asymptomatic individuals ranging in age from 36 to 65 years old (average age, 53 ± 17 years), with no significant alterations in electrocardiography, chest x-ray, echocardiogram, esophagogram and barium enema. All CARD patients (n = 10), ranging in age from 35 to 69 years (average age, 52 ± 17 years), presented dilated cardiomyopathy, characterized by the echocardiographic finding of a dilated left ventricle with impaired ventricular systolic function, which were classified as belonging to the group CARD V, as previously reported [7]. Left ventricular end-diastolic diameter/body surface area ≥31mm (64.8 ± 5.9mm) and left ventricular ejection fraction <55% (34 ± 10%) were used as echocardiographic parameters of Chagas dilated cardiomyopathy. Six normal healthy individuals, ranging in age from 30 to 49 years (average age, 39 ± 9 years) and showing negative serological tests for the infection, were from a non-endemic area for Chagas disease and were included as a control group (non-infected [NI]).

### Ethics statement

Written informed consent was obtained from all individuals prior to their inclusion in the study. Independent of their participation in this study, all individuals enrolled were submitted to a standard screening protocol, follow up and clinical treatment. This study was carried out in full accordance with all International and Brazilian accepted guidelines and was approved by the Ethics Committee at René Rachou Research Center-FIOCRUZ/MG (15/2011 CEPSH-IRR).

### *T*. *cruzi* antigens

Tissue culture-derived trypomastigotes of *T*. *cruzi* (Y strain) was used for soluble trypomastigote antigen (TRYPO). Parasites were subjected to rupture and homogenization in cold phosphate-buffered saline (PBS-Sigma-Aldrich, St. Louis, MO, USA), using a glass homogenizer and Teflon pestle, on ice to prevent overheating. Subsequently, the suspensions were centrifuged at 23.000g for 60 minutes at 4°C. The supernatant was collected, dialyzed for 24 hours at 4°C against phosphate-buffered saline (PBS), and sterilized by filtration on 0.2μm-pore-size membranes. The protein concentration was measured and the material was separated into aliquots and stored at -70°C until use.

### Flow cytometry analysis of peripheral blood *ex vivo*

Whole blood was collected in Vacutainer tubes containing EDTA (Becton, Dickinson, USA) and 100μL aliquots were mixed in tubes with 2μL of undiluted monoclonal antibodies anti-CD14 (clone MΦP9) conjugated with peridinin chlorophyll protein complex (PerCP) (BD Pharmingen^™^, USA). After erythrocyte lysis, cells were washed, permeabilized and incubated with monoclonal antibodies against MMP-2 (1A10) and MMP-9 (56129) (R&D Systems, USA) and IL-1β (AS10), TNF-α (6401.1111), TGF-β (9016) or IL-10 (JES3-9D7) (BD Pharmingen^™^, USA) conjugated with phycoeritrin (PE) or fluorescein isothiocyanate (FITC). After incubation, cells were fixed, and phenotypic analyses were performed by flow cytometry using FACSCalibur cytometer (BD Biosciences, Breda, Netherlands). A total of 7x10^4^ neutrophils and monocytes (gated based on forward and side scatter plot) were collected and data were analyzed using FlowJo software. Neutrophils and monocytes analysis was performed using a dot plot of anti-CD14 (FL3)/SSC. The neutrophils were gated as SSC^High^CD14^low^ and monocytes were gated as SSC^low^CD14^High^ [30].

### Whole blood cultures

A 5mL sample of peripheral blood was collected by venipuncture from each subject using a sterile Vaccutainer tube containing heparin as anticoagulant. Aliquots of 1mL of whole blood were mixed with TRYPO diluted in RPMI-1640 medium at final concentration of 20μg/mL. Aliquots of 1mL of whole blood were mixed with 1mL of RPMI-1640 medium for culture control. The samples were incubated for 18 hours in CO_2_ incubator with 5% humidity at 37°C, followed by addition of Brefeldin A (1mg/mL). The incubation continued for 4 hours under the same conditions, total of 22h of incubation. After incubation, 200μL of EDTA at a final concentration of 2mM were added directly to cultures and samples were incubated for another 15 minutes, followed by washing with PBS. The phenotype analysis of both surface and intracellular molecules was performed as described above.

### Assessment of inflammatory/regulatory balance and overall profile

We calculated a global median for neutrophils and monocytes MMPs^+^ and cytokines^+^ as a cut-off, using the data of flow cytometry analysis from all groups (NI, IND and CARD) after TRYPO stimulation, as previously reported by Vitelli-Avelar et al. and Luiza-Silva et al. [33, 34]. High inflammatory producers were classified when values of frequency of MMP-9, IL-1β and TNF-α cells^+^ are above median. High regulatory producers were defined when the values of MMP-2, TGF-β and IL-10 are above median. Low producers were defined when the values are below median for all molecules evaluated. The data is depicted in a color diagram representing low-producers (white square for all cytokines and MMPs), high inflammatory molecules-producers (black square for MMP-9, IL-1β and TNF-α) and high regulatory molecules-producers (gray square for MMP-2, TGF-β and IL-10) within each cell subset. We show diagrams for each patients, group and molecules, and a total line that were used to calculate an overall profile. Pie charts were used to represent the percentage of neutrophils and monocytes that were classified in low, high inflammatory, high regulatory or mixed (black/white squares) producers. The overall profile of MMPs or cytokine from neutrophils and monocytes was constructed by giving the same weight to all MMPs or cytokines and producing cell populations.

### Statistical analysis

Statistical analyses were performed using GraphPad Prism 5.0 software package (San Diego, CA, USA). All data files assume a non-Gaussian distribution and statistical comparisons were carried out using the nonparametric Kruskal-Wallis test, followed by Dunn's multiple comparison test for NI, IND and CARD groups. Comparative evaluation between *ex vivo* analysis and *in vitro* stimulated culture were performed by Wilcoxon matched pairs test. The association between MMPs and cytokines was performed by linear regression using the coefficient of determination (R^2^) to measure the goodness of fit and the *F* test to compare variances. Correlation analysis was done using Spearman's correlation coefficient by JMP software (Cary, NC, USA). In all cases, significance was considered at p<0.05.

## Results

### MMPs expression is increased in neutrophils and monocytes from patients with Chagas disease

**Fig 1:**
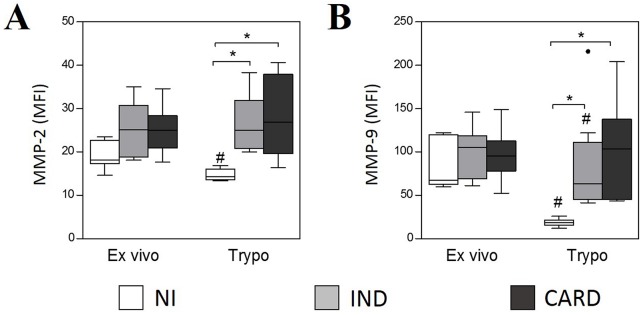
Intracytoplasmic levels of MMP-2 (A) and MMP-9 (B) in neutrophils SSC^high^CD14^low^. The analysis was performed as described in Materials and Methods. MMPs expression was measured by mean fluorescence intensity (MFI). The groups evaluated were NI (n = 6), IND (n = 8) and CARD (n = 10). Significant differences (p<0.05) in the charts are identified by asterisks (*) and connecting lines for comparisons between the groups and by the pound sign (#) for paired comparison analysis between *ex vivo* and stimulated culture with TRYPO.

Neutrophils from IND and CARD groups showed higher intracellular levels of MMP-2 (Fig 1A) and MMP-9 (Fig 1B) than NI group after *in vitro* stimulation with TRYPO. We did not observe statistical difference between neutrophils in *ex vivo* condition for MMP-2 (Fig 1A) and MMP-9 (Fig 1B) for all groups evaluated. However, when comparing MMPs expression before and after *in vitro* stimulation, TRYPO reduced the expression of MMP-2 by neutrophils from NI group (Fig 1A) and the expression of MMP-9 by neutrophils from NI and IND groups (Fig 1B).

**Fig 2:**
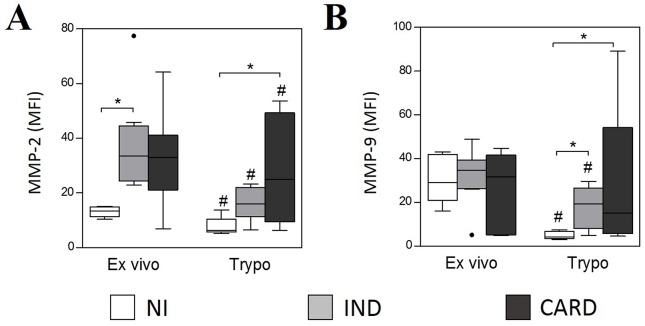
Intracytoplasmic levels of MMP-2 (A) and MMP-9 (B) in monocytes SSC^low^CD14^high^. The analysis was performed as described in Materials and Methods. MMPs expression was measured by mean fluorescence intensity (MFI). The groups evaluated were NI (n = 6), IND (n = 8) and CARD (n = 10). Significant differences (p<0.05) in the charts are identified by asterisks (*) and connecting lines for comparisons between the groups and by the pound sign (#) for paired comparison analysis between *ex vivo* and stimulated culture with TRYPO.

Monocytes from IND group showed higher expression of MMP-2 (Fig 2A) compared to NI group in *ex vivo* context. After *in vitro* stimulation with TRYPO, the expression of MMP-9 was higher in monocytes from IND group, while monocytes from CARD group presented higher expression of both MMP-2 and MMP-9 when compared to NI group (Fig 2A and 2B). As observed for neutrophils, *in vitro* stimulation with TRYPO induced a decrease of MMP-2 expression by monocytes in all groups and a decrease of MMP-9 expression by monocytes from NI and IND groups compared to *ex vivo* cells (Fig 2A and 2B).

Thus, *T*. *cruzi* derived-antigens promote a differential expression of MMPs in patients with Chagas disease and may regulate MMPs expression in neutrophils and monocytes, mainly when a cardiac alteration is not present. In addition, our data showed a higher expression of MMP-9 compared to MMP-2 in neutrophils and monocytes in all groups analyzed, despite of *in vitro* stimulation (S1 Fig).

### Monocytes appear to express cytokines mostly than neutrophils

**Fig 3:**
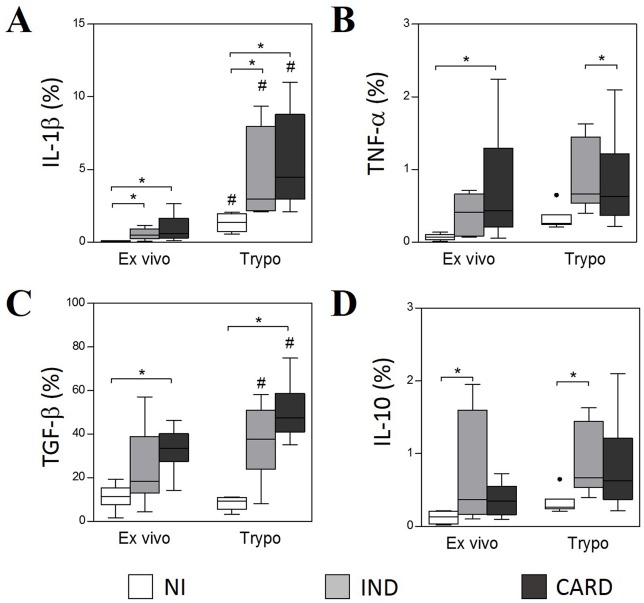
Intracytoplasmic levels of IL-1β (A), TGF-β (B), TNF-α (C) and IL-10 (D) in neutrophils SSC^high^CD14^low^. The analysis was performed as described in Materials and Methods. Cytokines expression was measured by percentage of positive cells. The groups evaluated were NI (n = 6), IND (n = 8) and CARD (n = 10). Significant differences (p<0.05) in the charts are identified by asterisks (*) and connecting lines for comparisons between the groups and by the pound sign (#) for paired comparison analysis between *ex vivo* and stimulated culture with TRYPO.

The expression of IL-1β was significantly higher in neutrophils from patients with Chagas disease compared to NI group, before and after *in vitro* stimulation. When stimulated with TRYPO, neutrophils from all groups presented an increase in the expression of IL-1β compared to *ex vivo* cells (Fig 3A). TNF-α expression was higher in neutrophils from CARD group in *ex vivo* context. However, after *in vitro* stimulation, this difference is no longer present and indeed a shift is observed, with neutrophils from IND group presenting higher TNF-α expression compared to CARD group (Fig 3B). Neutrophils from CARD group also showed higher expression of TGF-β than NI group in both, *ex vivo* and after *in vitro* stimulation. Moreover, TRYPO induced an increase in TGF-β expression in neutrophils from patients with Chagas disease when compared to *ex vivo* (Fig 3C). The expression of IL-10 by neutrophils from IND group was significantly higher compared to NI group, in *ex vivo* and after *in vitro* stimulation (Fig 3D).

**Fig 4:**
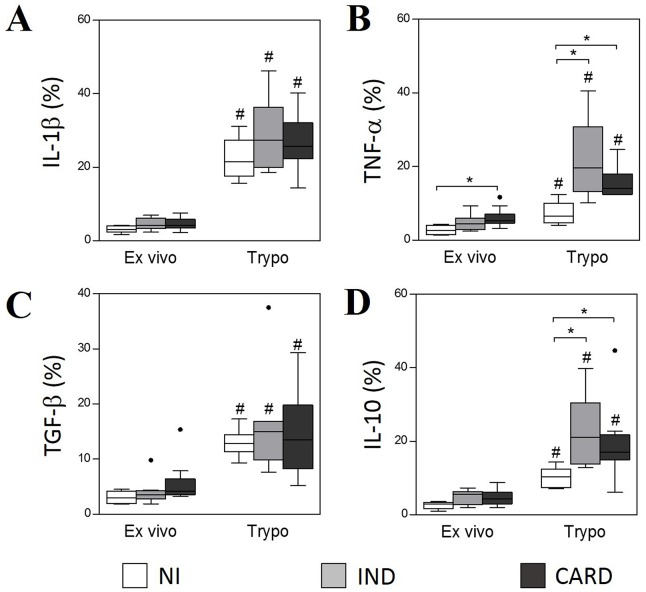
Intracytoplasmic levels of IL-1β (A), TGF-β (B), TNF-α (C) and IL-10 (D) in monocytes SSC^low^CD14^high^. The analysis was performed as described in Materials and Methods. Cytokines expression was measured by percentage of positive cells. The groups evaluated were NI (n = 6), IND (n = 8) and CARD (n = 10). Significant differences (p<0.05) in the charts are identified by asterisks (*) and connecting lines for comparisons between the groups and by the pound sign (#) for paired comparison analysis between *ex vivo* and stimulated culture with TRYPO.

The expression of these cytokines was also assessed in monocytes and the results showed that, in the presence of TRYPO, there is a significant increase in the percentage of monocytes expressing IL-1β, TNF-α, TGF-β and IL-10 in all groups (Fig 4). Our results showed no difference in the expression of IL-1β and TGF-β between the groups in *ex vivo* and after *in vitro* stimulation (Fig 4A and 4C). The expression of TNF-α in the *ex vivo* context was higher in monocytes from CARD group compared to NI (Fig 4B). After *in vitro* stimulation, monocytes from IND and CARD groups displayed a significant increase in the expression of TNF-α and IL-10 expression, compared to NI group (Fig 4B and 4D).

Our data show that in the presence of *T*.*cruzi* derived-antigens the production of cytokines by neutrophils and monocytes may be intensified. Although both cell lines are import sources for cytokines, we observed a higher frequency of monocytes (Fig 4) expressing cytokines compared to neutrophils (Fig 3) in *ex vivo* condition and after TRYPO stimulation.

### Antagonistic relationship between MMP-2 and MMP-9 with inflammatory and regulatory cytokines

**Fig 5:**
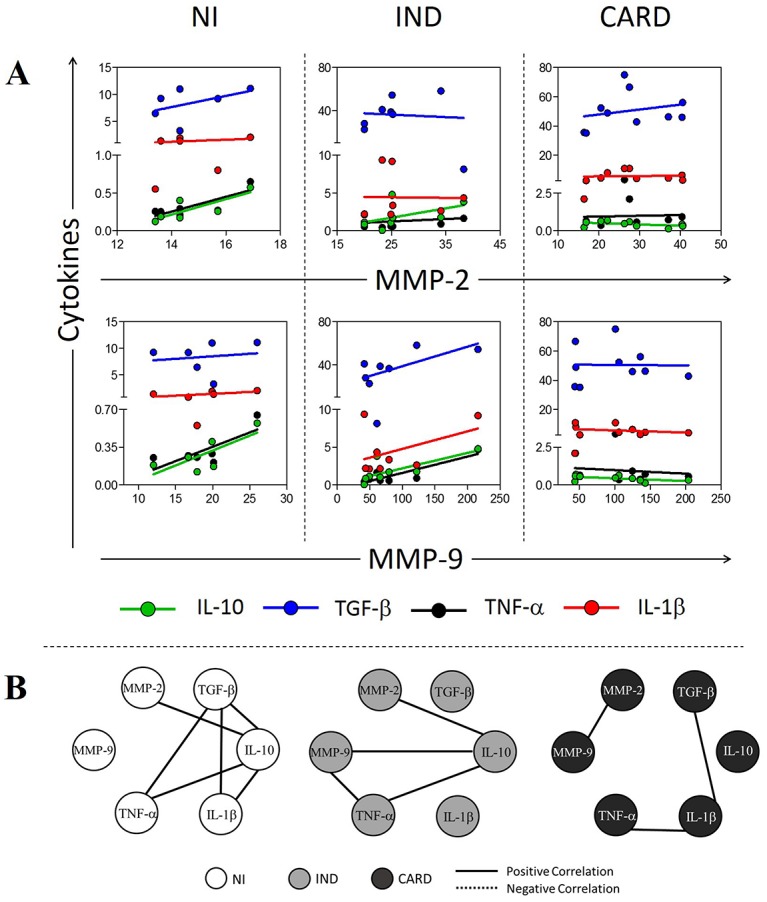
Association/Correlation analysis between MMPs and cytokines in neutrophils after *in vitro* stimulation with *T*. *cruzi* antigens. (A) Scatter plot with regression line between MMPs and cytokines. (B) Correlation network between MMPs and cytokines. All correlations shown in networks are strong, present ρ superior to 0.63 and statistical significance defined by p<0.05. The groups evaluated were NI (white circle n = 6), IND (light gray circle n = 8) and CARD (dark gray circle n = 10). The continuous lines represent positive correlation and traced lines represent negative correlation.

The linear regression analysis (Fig 5A and S1 Table) showed that MMP-2 expressed by neutrophils from NI group is significantly associated with IL-10 (R^2^ = 0.65, p<0.05) and TNF-α expression (R^2^ = 0.66, p<0.05). MMP-9 expressed by neutrophils from IND group is also associated with IL-10 (R^2^ = 0.56, p<0.05) and TNF-α expression (R^2^ = 0.80, p<0.05) (Fig 5A). MMPs expressed by neutrophils from CARD group had no significant association with cytokines by coefficient of determination (Fig 5A and S1 Table). Correlation analysis demonstrates how MMPs are associated with cytokines and cytokines between themselves. Our data showed that MMP-2 expressed by neutrophils have a positive correlation with IL-10 in NI (ρ = 0.81, p<0.05) and IND (ρ = 0.72, p<0.05) groups (Fig 5B). Moreover, MMP-9 expressed by neutrophils have a positive correlation with IL-10 (ρ = 0.81, p<0.05) and TNF-α (ρ = 0.71, p<0.05) in IND group (Fig 5B). Neutrophils from CARD group showed a positive correlation between MMP-2 and MMP-9 (ρ = 0.7091, p<0.05) expression (Fig 5B). IL-10 produced by neutrophils from NI group have a positive correlation with IL-1β (ρ = 0.83, p<0.05), TGF-β (ρ = 0.89, p<0.05) and TNF-α (ρ = 0.83, p<0.05); a positive correlation is also observed between TGF-β and IL-1β (ρ = 0.83, p<0.05), TGF-β and TNF-α (ρ = 0.83, p<0.05) (Fig 5B). Neutrophils from IND group showed a positive correlation between IL-10 and TNF-α (ρ = 0.93, p<0.05) (Fig 5B). Neutrophils from CARD group showed positive correlations between IL-1β and TNF-α (ρ = 0.72, p<0.05), IL-1β and TGF-β (ρ = 0.74, p<0.05) (Fig 5B).

**Fig 6:**
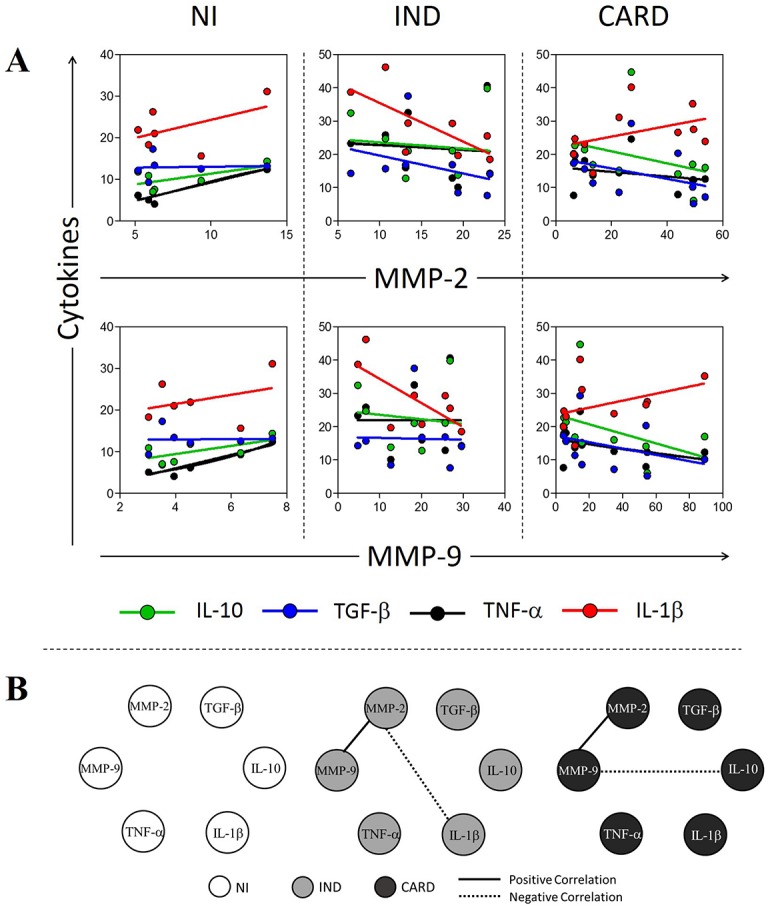
Association/Correlation analysis between MMPs and cytokines in monocytes after *in vitro* stimulation with *T*. *cruzi* antigens. (A) Scatter plot with regression line between MMPs and cytokines. (B) Correlation network between MMPs and cytokines. All correlations shown in networks are strong, present ρ superior to 0.63 and statistical significance defined by p<0.05. The groups evaluated were NI (white circle n = 6), IND (light gray circle n = 8) and CARD (dark gray circle n = 10). The continuous lines represent positive correlation and traced lines represent negative correlation.

For monocytes, MMP-2 (R^2^ = 0.86, p<0.01) and MMP-9 (R^2^ = 0.84, p<0.01) expression is significantly associated with TNF-α expression in NI group (Fig 6A and S1 Table). MMP-2 expressed by monocytes from IND group is significantly associated with IL-1β expression (R^2^ = 0.51, p<0.05) (Fig 6A and S1 Table). MMP-9 expressed by monocytes from IND and CARD groups had no significant association with cytokines by coefficient of determination (Fig 6A and S1 Table), as well as MMP-2 expressed by monocytes from CARD group (Fig 6A and S1 Table). MMP-2 expressed by monocytes from IND group showed a significant negative correlation with IL-1β (ρ = −0.78, p<0.05) (Fig 6B), while monocytes from CARD group showed a negative correlation between MMP-9 and IL-10 (ρ = −0.64, p<0.05) (Fig 6B). Monocytes from CARD (ρ = 0.90, p<0.05) and IND (ρ = 0.83, p<0.05) groups also showed a positive correlation between MMP-2 and MMP-9 expression (Fig 6B). Between the cytokines, no correlations were observed for monocytes from all groups studied (Fig 6B).

### Deficient regulation of inflammation may contribute with the development of Chagas cardiomyopathy

**Fig 7:**
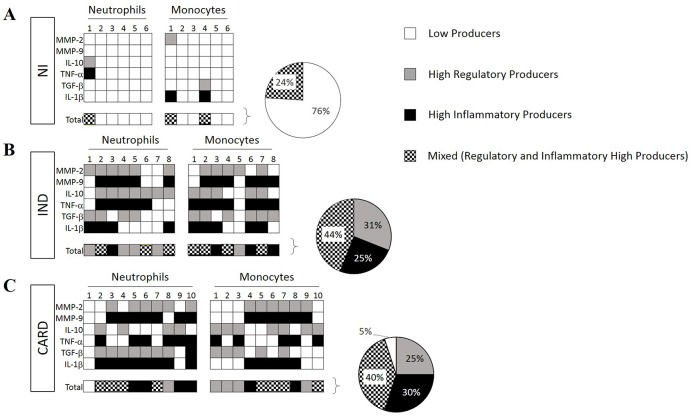
Analysis of the profile of low and high producers. Diagrams represent low (white), high inflammatory (black) high regulatory (gray) and mixed (black and white) producers for neutrophils and monocytes from chagasic patients (IND, n = 8 and CARD, n = 10) and non-infected individuals (NI, n = 6).

Our data showed that neutrophils and monocytes from IND and CARD groups were predominantly higher MMP and cytokine producers when compared to NI group (Figs 7 and S2). The results show that 76% of neutrophils and monocytes from NI group are low producers for all molecules evaluated, and 24% of these cells are compromised with mixed inflammatory and regulatory molecules (Fig 7A). On the other hand, 25% of neutrophils and monocytes from IND are classified as high inflammatory producers, while 31% of these cells are compromised with regulatory molecules (Fig 7B). Opposite profile was observed in CARD group, where 30% of neutrophils and monocytes are classified as high inflammatory producers, 25% are compromised with regulatory molecules and only 5% of neutrophils and monocytes from CARD group are characterized as low producers (Fig 7C). Approximately 40% of neutrophils and monocytes from IND and CARD groups presented a mixed profile, which means a balance between high-inflammatory and regulatory-cytokine producers (Fig 7B and 7C).

## Discussion

The structural basis for the development of cardiomyopathy, a severe consequence of Chagas disease, is the myocardial remodeling process [6, 35]. Both cellular and extracellular factors are involved in the remodeling process and a combined action of these factors promotes changes in myocardial structure [36, 37]. Our results showed that the expression of MMPs by neutrophils is higher than by monocytes, and indicate neutrophils as an important source of MMPs in Chagas disease, suggesting that these cells and MMPs may be involved in physiopathology of morbidity. Recent evidence suggests that neutrophils and monocytes can contribute significantly to the immune response by modulating both cellular and humoral immunity, especially in the synthesis and release of inflammatory and regulatory molecules [32, 38].

The participation of MMP-2 and MMP-9 in several heart diseases has been well established. Polyakova et al. [20] showed that elevated expression of MMP-2 and MMP-9 is associated with collagen maturation in heart failure, demonstrating an important role of these enzymes in fibrosis through collagen configuration, activation, and deposition. In experimental models of Chagas disease, high MMP-2 and -9 proteolytic activity has been described, which suggests an important role of MMPs in *T*. *cruzi*-induced acute myocarditis [21]. In agreement with these data, our results also demonstrated an increased production of MMP-2 and MMP-9 by neutrophils and monocytes from IND and CARD groups.

Despite the increase of MMPs in neutrophils and monocytes from infected patients compared to non-infected individuals, *T*. *cruzi*-derived antigens appears to regulate MMPs expression in these cells, mainly when a cardiac alteration is not present. These data reinforce that other mediators can influence the MMP expression during cardiac remodeling caused by Chagas disease. Indeed, our current findings demonstrated an association between MMP-2 and MMP-9 with cytokines, however with different correlations profile. We have shown a positive correlation between MMP-2 and IL-10 in neutrophils from NI and IND group, also a negative correlation between IL-1β and MMP-2 in monocytes. In contrast, MMP-9 showed a negative correlation with IL-10, a regulatory cytokine, in monocytes from CARD group, suggesting an antagonistic function between the MMPs studied. Whereas MMP-2 seems to be involved in regulation of the immune response, MMP-9 appears to be related to inflammation and development of Chagas cardiomyopathy. The suppressive role of MMP-2 and a pivotal role of MMP-9 in the development of inflammatory joint disease was described in rheumatoid arthritis [39] and other studies support this antagonistic role of MMP-2 and MMP-9 in inflammation [13, 39, 40].

It has been demonstrated that MMPs are intimately involved in the regulation of cytokine function and cytokine receptor expression [18, 26, 41, 42]. MMP-2 and MMP-9 are capable to degrade IL-1β into biologically inactive fragments, but on the other hand, these proteins are also capable to cleave IL-1β precursor molecules into biologically active forms. Thus, MMPs can both up- and down-regulate cytokine activities at sites of acute or chronic inflammation [41, 42]. Schönbeck et al. showed that IL-1β can be activated in an alternative pathway that involves MMP-9 [42]. Indeed, IL-1β is one of the classical inducers of MMPs, including MMP-2 and MMP-9, in different cell types, leading to a mechanism of positive feedback [43, 44]. Here, we demonstrated the significant association between IL-1β and MMP-2 by monocytes, yet the correlation between these molecules was negative. Once again, MMP-2 seems to be poorly involved in inflammation.

Latent TGF-β can also be activated by MMP-2 e MMP-9 and this may provide a physiological mechanism of tissue remodeling [45, 46]. Besides the role of TGF-β in regulating inflammation and outcome of many infections, this cytokine plays a relevant role in cardiovascular development, physiology and disease, with a predictable important effect in heart fibrosis, through activation of MMPs, deposition of MEC and differentiation of fibroblasts [47, 48]. Moreover, MMP-2 and MMP-9 can activate latent TGF-β in a two-way traffic [49, 50]. We showed an increase of TGF-β after *in vitro* stimulation with TRYPO suggesting that this cytokine may contribute for the development of Chagas disease. Furthermore, high intracytoplasmic levels of TGF-β in neutrophils from CARD group also indicate the participation of this cytokine in cardiac remodeling and MMPs production in chagasic cardiomyopathy.

Cytokine production contributes for matrix remodeling and inflammatory diseases. Indeed, the overall cytokine profile seems to confirm these observations. Our results showed that despite the similar inflammatory profile in IND and CARD patients, IND group has a greater and more efficient immune regulation, indicating that this is the key point to prevent the development of heart disease. The present study investigated neutrophils and monocytes as MMPs and cytokines producers, and the results showed that IND group has major quantity of high producers of regulatory molecules, such as MMP-2, TGF-β and IL-10 that may promote a modulatory effect on inflammatory molecules like MMP-9, TNF-α and IL-1β. In contrast, the CARD group showed an increase of high producers of inflammatory mediators, such as MMP-9, TNF-α and IL-1β that may be related to the development of cardiac changes and exacerbation of inflammation.

The results also demonstrated the loss of important correlations in neutrophils from CARD group compared to NI and IND, mainly the interactions involving IL-10 with MMP-2 and TNF-α. Cytokines produced by neutrophils from NI presented positive correlations to each other, suggesting a possible equilibrium in the immune response. Those correlations seem to be disorganized in infected patients, mostly in CARD patients. Campi-Azevedo et al. also described a similar immune response profile in infected patients, where CARD group showed the loss of important interactions [51]. These results taken together may indicate a balanced profile between MMPs and cytokines produced by neutrophils and monocytes from individuals without cardiac alteration.

Thus, these findings suggest that MMPs are differentially involved in Chagas’ cardiomyopathy, whence MMP-2 are correlated with regulatory cytokines and MMP-9 with inflammatory cytokines, suggesting an antagonistic relationship in inflammation. MMP-2 appears to be related to cardiac-protective and regulatory functions in IND group, while predominance of MMP-9 can be identified as an inflammatory booster, favoring the development of Chagas cardiomyopathy. In conclusion, our results emphasize at least three important functions of neutrophils and monocytes in chronic phase of Chagas disease: 1) Orchestrate an adaptive response; 2) Participate in the remodeling of heart ECM, through the production of MMP-2 and MMP-9; 3) Contribute to the maintenance or regulation of inflammation via the production of distinct cytokines.

## Supporting Information

S1 Fig MMP-2/MMP-9 balance in neutrophils (A) and monocytes (B) in *ex vivo* and *in vitro* stimulation. The groups evaluated were NI (n = 6), IND (n = 8) and CARD (n = 10). The graphic was built from median values of intracytoplasmic levels of MMPs in flow cytometry analysis.
(TIF)

S2 Fig Representative scatter plot used to establish the cut-off to define low and high producers. Traced lines represent the median for all groups (NI, n = 6; IND, n = 8; CARD, n = 10). Low producers were defined by values lower than median and high producers were defined by values higher or equal to median. The numbers indicate the percentage of high producers cells in each group.
(TIF)

S1 Table Linear regression between MMPs and Cytokines. (XLSX)
