# Supplementary figures and images for: Differential Expression of Matrix Metalloproteinases 2, 9 and Cytokines by Neutrophils and Monocytes in the Clinical Forms of Chagas Disease
Source: PLoS Negl Trop Dis. 2017 Jan 24;11(1):e0005284. doi: 10.1371/journal.pntd.0005284 (PMC5261563; doi:10.1371/journal.pntd.0005284)

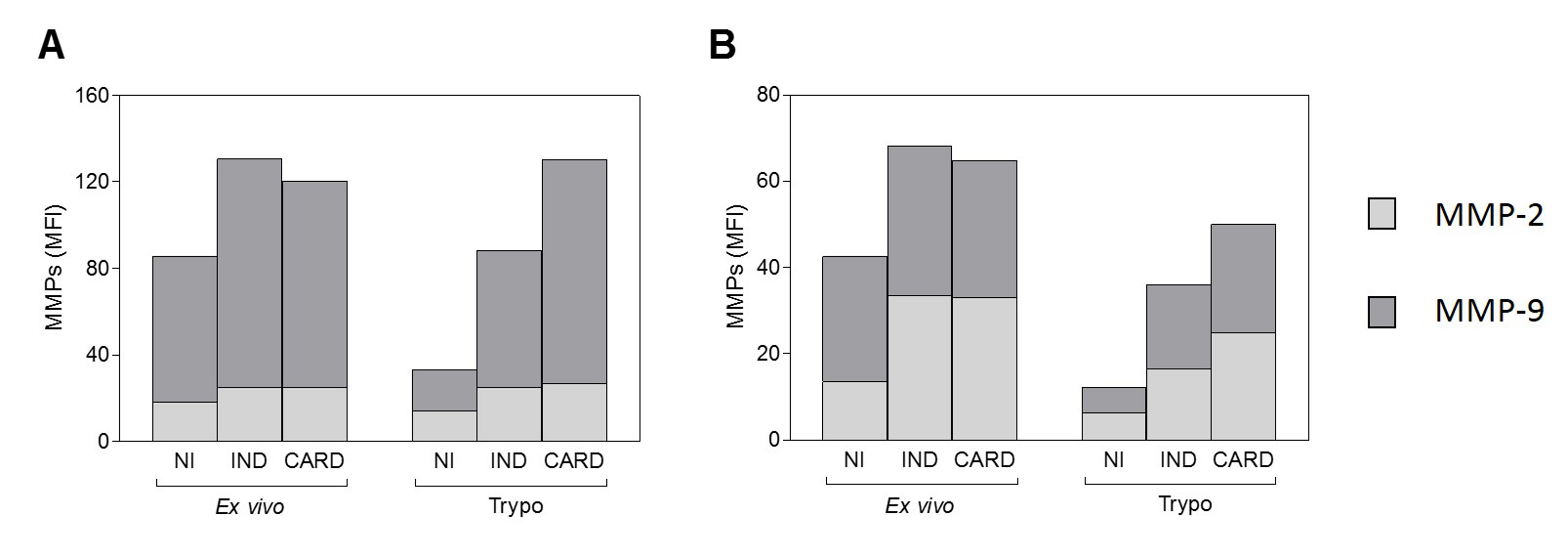

Supplement: S1 Fig — The groups evaluated were NI (n = 6), IND (n = 8) and CARD (n = 10). The graphic was built from median values of intracytoplasmic levels of MMPs in flow cytometry analysis. (TIF) [file pntd.0005284.s001.tif]

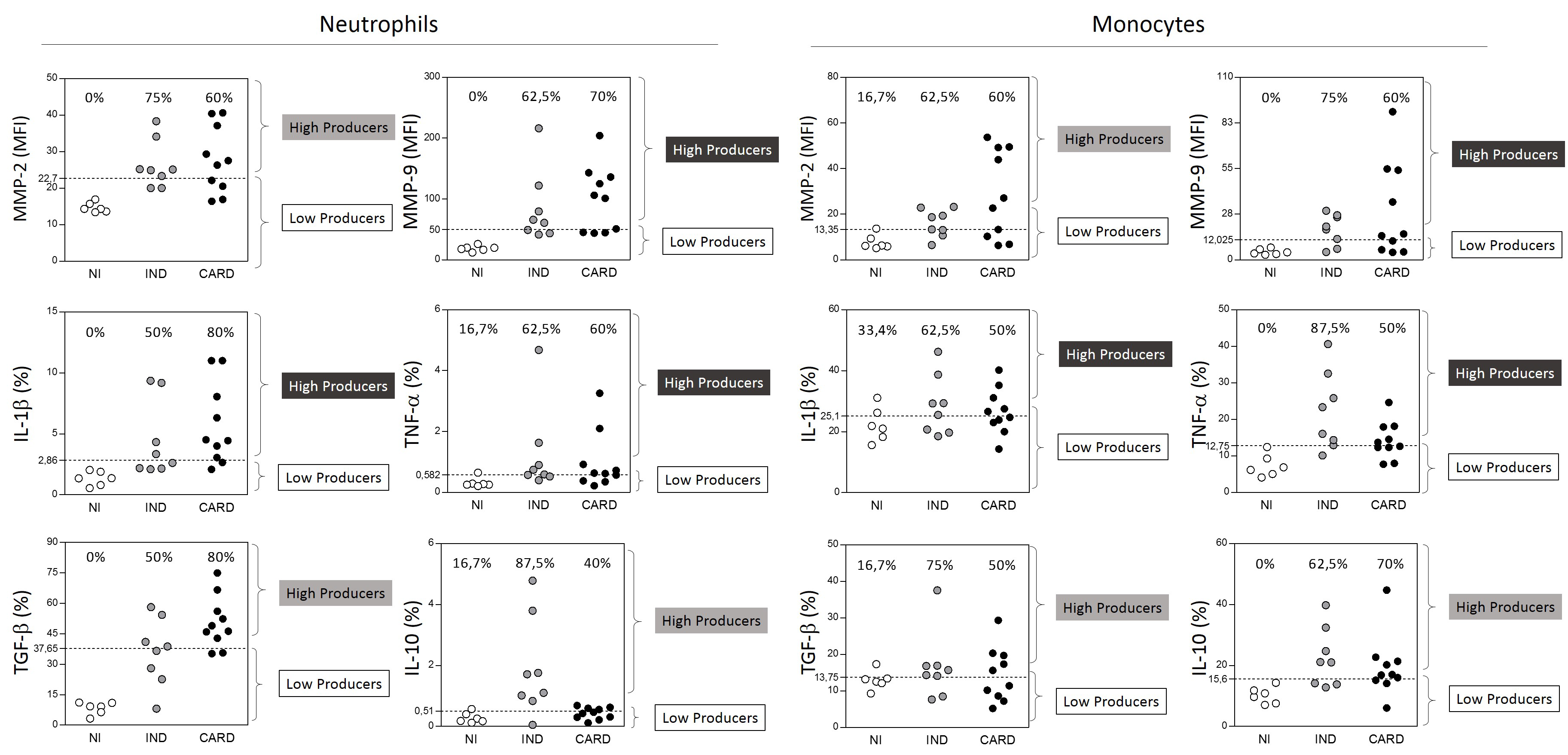

Supplement: S2 Fig — Traced lines represent the median for all groups (NI, n = 6; IND, n = 8; CARD, n = 10). Low producers were defined by values lower than median and high producers were defined by values higher or equal to median. The numbers indicate the percentage of high producers cells in each group. (TIF) [file pntd.0005284.s002.tif]
